# Elinzanetant improves sleep in postmenopausal women. So does placebo

**DOI:** 10.1093/sleep/zsag210

**Published:** 2026-07-28

**Authors:** Julia M Lappin, Aurore A Perrault, Christopher J Gordon

**Affiliations:** Discipline of Psychiatry and Mental Health, School of Clinical Medicine, UNSW Sydney, Sydney, New South Wales, Australia; Sleep & Circadian Research Group, Woolcock Institute of Medical Research, Macquarie University, Sydney, New South Wales, Australia; Optimizing Brain Health from Midlife Clinic, Neuroscience Research Australia, Randwick, Sydney, New South Wales, Australia; Sleep & Circadian Research Group, Woolcock Institute of Medical Research, Macquarie University, Sydney, New South Wales, Australia; Sleep & Circadian Research Group, Woolcock Institute of Medical Research, Macquarie University, Sydney, New South Wales, Australia; School of Health Sciences and Nursing, Faculty of Medicine, Health and Human Sciences, Macquarie University, Sydney, New South Wales, Australia

Baker and co-authors’ well-conducted multi-center trial utilizing both subjective and objective measures of sleep provides evidence that elinzanetant reduces sleep difficulties and improves quality of sleep in postmenopausal women with moderate to severe vasomotor symptoms (VMS) [1]. A strong placebo response, however, resulted in elinzanetant’s superiority at 4 weeks being rendered no longer statistically significant at 12 weeks. This raises a number of questions, including what the clinical significance of elinzanetant may be. Here, we consider why postmenopausal sleep disturbances warrant special attention and the rationale for elinzanetant in their treatment. We highlight known unknowns regarding elinzanetant, including whether its effects are (only) via VMS and/or via other mechanisms. We note the common overlap between sleep and mood disturbances in this population and discuss the strong placebo response observed here, consistent with strong placebo responses reported in both sleep and mood research.

Sleep disturbance associated with the menopause is experienced by two in three women and significantly impacts quality of life, relationships, and work performance [2]. Effective treatments are much needed, as while menopausal hormone therapy (MHT) reduces VMS and indirectly benefits sleep [3], it is not indicated for sleep disturbances alone and for some women is contraindicated. Unique characteristics of perimenopausal (symptoms in the up to 10 years before total cessation of menstrual periods, i.e. menopause) and postmenopausal women’s sleep disturbances include objective increases in both wake after sleep onset (WASO) and number of nighttime awakenings, making these appropriately-selected outcomes in this comprehensive RCT [1]. Increasing prevalence of self-reported awakenings was the sleep problem which contributed most strongly to the increasing prevalence of overall sleep difficulties observed from several years prior to menopause through several years postmenopause in the SWAN study, a large, well-conducted longitudinal study observing women throughout the menopause transition [4]. Another key difference between peri/postmenopausal sleep disturbances and those reported in insomnia disorder in other populations is that early morning awakenings and trouble falling asleep are less commonly reported: while 40 per cent of women in SWAN complained of awakenings, only 20 per cent reported early morning awakenings and 20 per cent trouble falling asleep [4], findings replicated in other peri/postmenopausal cohorts [5, 6]. Several mechanisms have been posited to underlie peri/postmenopausal sleep disturbances, including VMS (night sweats and hot flashes/flushes [HFs]), mood symptoms, and other issues including thermoregulatory changes beyond VMS alone [7].

Elinzanetant is an effective nonhormonal alternative for the management of VMS in women for whom MHT is contraindicated or undesirable [8]. A dual neurokinin NK-1-3 receptor antagonist, it is believed to act on hypothalamic estrogen-sensitive kisspeptin/neurokinin B/dynorphin (KNDy) neurons, with potential benefits on thermoregulation, mood, and circadian sleep–wake patterns [2, 7]. Estrogen fluctuations in the perimenopause segue into dramatic decreases postmenopause with concomitant hyperactivity of KNDy neurons and increased body temperature with, at times, cutaneous vasodilation and sweating giving rise to VMS. It is not yet known whether KNDy neuron hyperactivity additionally impacts other physiological/thermoregulatory changes which increase sleep disruption in peri/postmenopausal women.

Elinzanetant is known to be effective in reducing VMS, so one question which follows naturally is whether this is the mechanism through which elinzanetant-associated sleep improvements are achieved. This was not the focus of the current study [1], so it is unclear whether VMS are the only story here or whether other physiological/thermoregulatory factors are impacting sleep. Eliminating VMS alone would not be sufficient to resolve all awakenings among peri/postmenopausal women, as, on average, only 27 per cent of their time awake is HF-associated, and high variability exists between women in both the number of HF measured and the associated wakefulness [9]. To explore which elinzanetant-associated sleep improvements occur secondary to VMS improvement and which to other factors, future studies should examine sleep outcomes in peri/postmenopausal women with varying levels of VMS, including none. Further, it is unclear whether any other factors impacting sleep are specific to elinzanetant or whether they may be nonspecific effects also operating in the placebo group.

Improvement in placebo arms of RCTs is observed in both objective and subjective measures of sleep in men and women [10, 11] and in HFs in women [12]. There are parallels with other neuropsychiatric conditions, e.g. depression, which meta-analytic data show to have a very large effect size, equating to an up to 50 per cent reduction in symptoms [13]. There is increased prevalence of mood symptoms peri/postmenopausally, which are associated with sleep disruption [14, 15]. So, in peri/postmenopausal women, there is a high prevalence of comorbid sleep, VMS, and mood symptoms, all of which have a significant placebo response. Placebo response may be contributed to both by the placebo effect—factors including participants’ expectations, therapeutic alliance, and broader contextual factors which contribute to therapeutic benefit [16]. A putative contributor in this RCT [1] was the regular discipline of keeping a sleep diary and the potential positive effects that simply attending to/prioritizing their sleep might provide to participants. Also potentially relevant here, subjective reports of wakefulness are highly correlated with HF-associated waking, indicating that women are most aware of being awake during the wake periods associated with HF [9]. Further, objective and subjective reports of VMS are poorly correlated [17], so, arguably, believing that their HF may be improving could lead women’s subjective experiences of VMS, and thus of wakefulness, to decrease.

Placebo response is also contributed to by other factors which have been observed in previous sleep trials [18, 19] including the Hawthorne effect (changes in participant behavior or reporting because s/he is being observed), spontaneous recovery, and regression to the mean—the phenomenon where participants are selected at a time when their symptoms are more severe than average due to natural fluctuations which, with time, return/regress back to a normative level (reviewed in Yeung et al. [20]). It is possible that this factored into this RCT [21], where participants had baseline WASO values of 74.7 min, far exceeding the >30 min typically required in insomnia trials, and where 54.6 per cent had >50 moderate-to-severe VMS per week. Future research could employ a third no-treatment comparison group in which participants remain subject to these factors but not to the placebo effect itself [20].

Brain imaging and neurophysiological data show that placebo effects are associated with neurobiological substrates, supporting a model in which participant expectancies lead to modulation of brain networks and brain–body systems implicated in certain neuropsychiatric conditions such as depression and Parkinson’s disease [16]. Thus, there is overlap between the neural circuits activated by treatments for these conditions and those activated by placebo. So, it is not that the drugs don’t work, but rather that placebos works too. This has implications not only for research—indicating a need to consider and control for the therapeutic benefits of placebo—but also clinically, to utilize placebo effects alongside active treatments. Potentially therapeutic elements, such as allowing expectancies and prioritizing attending to one’s sleep, could be transferred effectively into clinical practice as potentially beneficial interventions [16].

In summary, Baker and coworkers [1] report the efficacy of elinzanetant in reducing WASO and awakenings/arousals, an important advance in identifying a treatment specifically targeting postmenopausal sleep disturbances. Future research should aim to elucidate the mechanisms underlying peri/postmenopausal sleep disturbances and those through which elinzenetant and other pharmacological treatments, including MHT, may exert their therapeutic effect, with acknowledgment of the contribution of placebo.
